# Incidence and lesions causative of delusional misidentification syndrome after stroke

**DOI:** 10.1002/brb3.1829

**Published:** 2020-09-07

**Authors:** Yasuro Kakegawa, Osamu Isono, Keisuke Hanada, Takashi Nishikawa

**Affiliations:** ^1^ Department of Clinical Rehabilitation Graduate School of Comprehensive Rehabilitation Osaka Prefecture University Habikino‐City Osaka Prefecture Japan; ^2^ Department of Rehabilitation Kyoto Min‐iren Asukai Hospital Kyoto‐City Kyoto Prefecture Japan; ^3^ Department of Neurology Kyoto Min‐iren Asukai Hospital Kyoto‐City Kyoto Prefecture Japan; ^4^ Department of Rehabilitation Suishokai Murata Hospital Osaka City Osaka Prefecture Japan

**Keywords:** delusional misidentification syndrome, Fregoli syndrome, somatoparaphrenia, stroke, uncinate fasciculus

## Abstract

**Objective:**

To better elucidate the symptomatology and pathophysiological mechanisms underlying delusional misidentification syndrome (DMS), we investigated the incidence rate and symptomatic features of DMS following stroke and relationships among DMS, other neuropsychological symptoms, and lesion locations.

**Methods:**

The present study included 874 consecutive patients (371 women; mean age ± standard deviation = 72.2 ± 11.7 years) who were admitted to the rehabilitation wards at two hospitals within 2 months of their first stroke. We examined the clinical features and lesion sites of patients with DMS and compared them with those of a control group of patients with hemi‐spatial neglect without DMS using voxel‐based lesion–symptom mapping (VLSM).

**Results:**

Among the 874 patients who experienced a stroke, we observed 10 cases of Fregoli syndrome. No other DMS subtypes were observed; however, eight patients exhibited somatoparaphrenia (five of them also had Fregoli syndrome) and one also exhibited reduplicative paramnesia. Right hemispheric lesions were found in all 10 cases. VLSM revealed statistically significant overlapping lesion sites specifically related to Fregoli syndrome when compared with the control group. The sites included the insula, inferior frontal lobe, anterior temporal lobe, and subcortical limbic system in the right hemisphere (i.e., areas connected by the uncinate fasciculus).

**Conclusion:**

The DMS incidence was 1.1% among patients after stroke. All patients had Fregoli syndrome and half had somatoparaphrenia, suggesting that the two syndromes share an underlying pathology. Lesions found with Fregoli syndrome were concentrated around the right uncinate fasciculus; this has not been reported in previous research.

## Graphical abstract

The incidence of DMS was 1.1% after stroke; all of these patients had Fregoli syndrome. The lesions were concentrated around the right uncinate fasciculus.

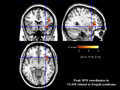

**Limitations**

We only evaluated patients from the rehabilitation wards of two hospitals and did not include data from patients in the acute phase of stroke or those who were living at home. No standardized assessment method for DMS has been established. More cases might have been detected if we had used other criteria.

## 1 INTRODUCTION

Delusional misidentification syndrome (DMS) refers to the delusional misidentification of other people (Christodoulou, 1986; Christodoulou & Malliara‐Loulakaki, 1981). According to its classification (Christodoulou, 1986), DMS has four subtypes: Capgras syndrome, which is characterized by the belief that a familiar person has been substituted with an imposter (Capgras & Reboul‐Lachaux, 1923); Fregoli syndrome, which is characterized by the belief that an acquaintance is disguised as another person with a different appearance (Courbon & Fail, 1927); intermetamorphosis, which is characterized by the belief that two known people have been switched (Courbon & Tusques, 1932); and subjective doubles, which is characterized by the belief that a double of oneself exists and is performing actions independently (Christodoulou, 1978). Although these syndromes were originally reported to be the products of psychoses, similar symptoms—especially those associated with Capgras and Fregoli syndromes—have been reported in cases of organic brain damage (Christodoulou, 1976; Darby, Laganiere, Pascual‐Leone, Prasad, & Fox, 2017; Darby & Prasad, 2016; Devinsky, 2009; Hirstein, 2010; Kakegawa, Isono, & Nishikawa, 2016). Because of their potential to evince the neural mechanisms underlying delusions, such cases have garnered increasing attention. Research has suggested that lesions in the right cerebral hemisphere and frontal lobe (bilateral) underlie Capgras and Fregoli syndromes. Research has also suggested that delusions are not caused by impaired perception or memory; instead, they may be related to impaired emotional judgment (Christodoulou, 1986; Christodoulou, Margariti, Kontaxakis, & Christodoulou, 2009). A lack of familiarity with the subject of delusion (hypo‐identification) is observed in Capgras syndrome, whereas excessive familiarity is observed with Fregoli syndrome (hyper‐identification).

To the best of our knowledge, the incidence of DMS among patients who have experienced a stroke has not been studied. Moreover, few studies (Darby & Prasad, 2016) have investigated the relationships among DMS, other neuropsychological symptoms, and the lesion site.

### 1.1 AIMS OF THE STUDY

To elucidate the pathophysiological mechanisms underlying delusional misidentification syndrome, the present study performed prospective observations of inpatients who had experienced a stroke at two hospitals over the course of 4 years.

## 2 MATERIALS AND METHODS

### 2.1 Participants

The present study included 874 consecutive patients (503 men and 371 women; mean age ± standard deviation [*SD*] = 72.2 ± 11.7 years) who were admitted to the rehabilitation wards at two hospitals within 2 months of their first stroke between August 2009 and November 2013. Patients with a history of multiple strokes, degenerative dementia, or psychiatric disorders at stroke onset were excluded.

### 2.2 Procedures

Medical doctors and rehabilitation staff who had already learned and shared the concept and classification of DMS by Christodoulou (1986) through in‐hospital lectures prospectively monitored all participants for any speech and attitude that would suggest the misidentification of individuals. To distinguish these symptoms from temporal illusions or cognitive dysfunction, diagnoses of DMS were confirmed only when such speech or behavior was confirmed at multiple time points.

When DMS was detected, patients were evaluated in order to determine the DMS subtype (Capgras syndrome, Fregoli syndrome, intermetamorphosis, or subjective doubles) and whether the presence of delusional misidentification for items other than people existed, such as reduplicative paramnesia or somatoparaphrenia. Patients’ neurological and neuropsychological complications were examined using the Mini‐Mental State Examination (MMSE) (Folstein, Folstein, & McHugh, 1975), Frontal Assessment Battery (FAB) (Dubois, Slachevsky, Litvan, & Pillon, 2000), and tests of unilateral spatial neglect (line cancellation, line bisection test, and graphic replication). The Visual Perception Test for Agnosia (VPTA) (Japan Society for Higher Brain Dysfunction Brain Function Test Committee, 2003) was also utilized to evaluate face recognition when prosopagnosia was suspected. The symptomatic features of DMS we evaluated included the misidentification of individual(s) or object(s), content of delusions, consistency of delusional statements, and the duration of delusions. The lesion location was examined using magnetic resonance imaging (MRI) in all cases. Each patient with DMS was monitored only during the hospitalization period.

### 2.3 Lesion analysis

To identify lesions that caused DMS, we created a control group of stroke patients without DMS, the details of which are given in the “Lesion locations” subsection of the Results. We compared the lesion locations in the DMS group with those in the control group.

A lesion analysis of the collected brain imaging data was performed. The AC‐PC line was set automatically using a custom MATLAB script. One of the authors who had no information about the patients’ symptoms delineated lesions with anonymized imaging scans using *Clusterize* with SPM12 (de Haan, Clas, Juenger, Wilke & Karnath, 2015). Those delineated lesions were spatially normalized to the Montreal Neurological Institute (MNI) template using the Clinical Toolbox with SPM12 (Rorden, Bonilha, Fridriksson, Bender, & Karnath, 2012). Finally, MRIcron software (http://www.mccauslandcenter.sc.edu/mricro/mricron/index.html) was used to construct images of overlapping lesions for the DMS and non‐DMS groups. Nonparametric mapping (NPM) implemented in MRIcron was used for the lesion analysis (Rorden, Karnath, & Bonilha, 2007). To detect the putative statistical differences in lesion patterns between patients with and without DMS, the Liebermeister test was performed using binomial data (DMS group vs. non‐DMS group) (http://people.cas.sc.edu/rorden/mricron/stats.html). The lesion analyses included only voxels that were commonly identified in at least 10% of all patients in the Fregoli and non‐DSM groups (i.e., in at least 4 patients).

Voxels were considered significant when they exceeded a statistical threshold of *p* < .05, corrected by the false discovery rate. Anatomical localization extracted by voxel‐based lesion–symptom mapping (VLSM) and subtracting lesions in the non‐DMS group from those of DMS group was identified using automatic anatomical labeling (Tzourio‐Mazoyer et al., 2002) implemented in MRIcron. In addition, to examine whether the lesion volume was associated with the manifestation of DMS, we compared the lesion volumes in the DMS and the non‐DMS groups using the Mann–Whitney *U* test.

### 2.4 Standard protocol approval, registration, and patient consent

This study was approved by the Ethics Committee of the Graduate School of Comprehensive Rehabilitation, Osaka Prefecture University (2016‐202). A family member of each participant provided written informed consent.

### 2.5 Data availability

Relevant data can be obtained from the corresponding author on request.

## 3 RESULTS

### 3.1 Incidences of each DMS subtype and related symptoms

Diagnoses among the patient population included cerebral infarction (*n* = 571), cerebral hemorrhage (*n* = 223), subarachnoid hemorrhage (*n* = 44), and other causes of stroke (*n* = 36), including infratentorial hemorrhage and infarction, subdural and epidural hematoma, and unidentified accidents. Lesions were observed in the right hemisphere in 294 patients, the left hemisphere in 387 patients, and bilaterally (in the cerebellum or brainstem) in 193 patients.

We detected DMS in 10 patients. All 10 of those DMS patients also had Fregoli syndrome. No other DMS subtypes (i.e., Capgras syndrome, intermetamorphosis, subjective doubles) were observed. In addition, we found related misidentification symptoms such as somatoparaphrenia in eight patients (five of whom also had Fregoli syndrome), reduplicative paramnesia (reduplication of the patient's daughter) in one patient who also exhibited Fregoli syndrome and somatoparaphrenia, and mirror sign in one patient who also had somatoparaphrenia. Except for somatoparaphrenia for paretic limbs, no delusional misidentifications of other objects or events were observed.

**FIGURE 1:**
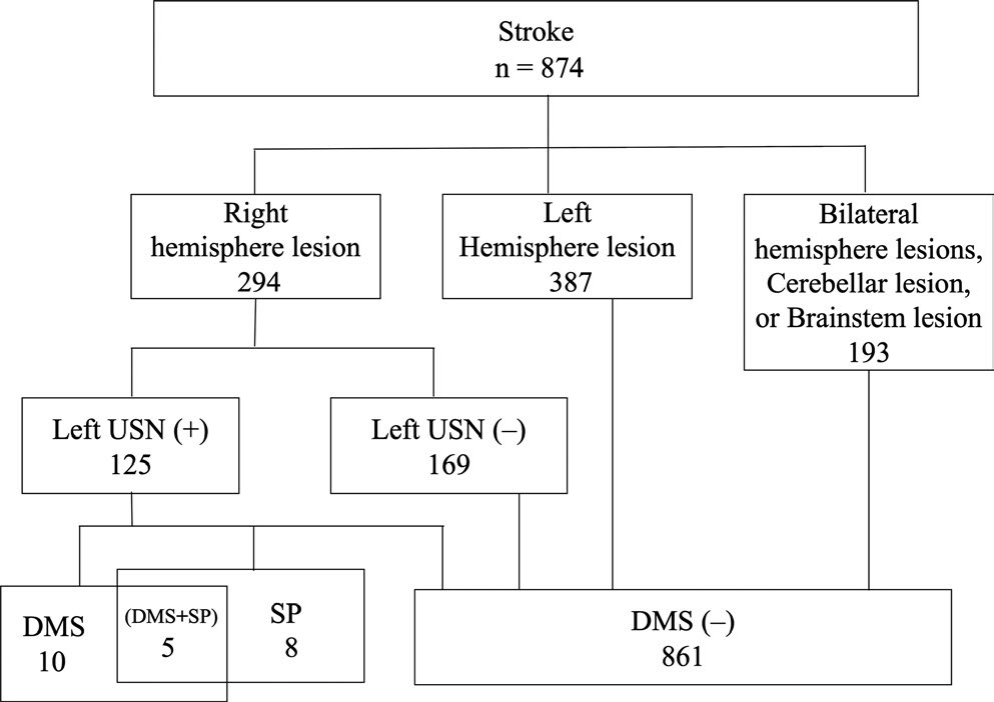
Incidence rate of DMS in patients after stroke. DMS, delusional misidentification syndrome; SP, somatoparaphrenia; USN, unilateral spatial neglect

Lesions of the right hemisphere were found in all 10 DMS patients with Fregoli syndrome (Figure 1). We did not observe DMS in patients with left hemispheric, bilateral hemispheric, or infratentorial lesions. Among the 294 patients with right hemispheric lesions, 125 patients exhibited left unilateral spatial neglect; all of the 10 DMS patients with Fregoli syndrome were included in this group of 125 patients. Therefore, the incidence rates of DMS were 1.1% among patients who had experienced a stroke, 3.4% among patients with right hemispheric lesions, and 8.0% among patients with left unilateral spatial neglect.

### 3.2 Neurological and neuropsychological complications and clinical findings

Table 1 summarizes the clinical findings for the 10 DMS patients with Fregoli syndrome. These 10 patients (3 women and 7 men) had an age range of 54–76 years (mean age ± *SD* = 67.9 ± 6.6 years). Diagnoses among these patients included cerebral infarction (*n* = 5), cerebral hemorrhage (*n* = 3), and cerebral infarction following subarachnoid hemorrhage (*n* = 2).

**TABLE 1:** Clinical findings for the ten patients with Fregoli syndrome

Patient	Age (years)	Sex	Diagnosis	Neurological impairments			Pusher syndrome	USN	Asomatognosia	Denial of paralysis	Visual agnosia		DMS		MMSE	FAB
				Paresis Br‐s	Superficial sensation	Proprioception					Object	Face	Person	Other		
1	61	M	Infarction	I–I–II	Severe	Severe	+	+	+	–	–	–	FS	–	19/30	–
2	67	M	Infarction	V–V–V	Mild	Mild	–	+	+	–	–	–	FS	–	29/30	17/18
3	64	M	Hemorrhage	I–I–I	Severe	Severe	+	+	+	+	–	–	FS	–	20/30	11/18
4	68	M	SAH, infarction	II− I–III	Severe	Severe	+	+	+	–	–	–	FS	–	16/30	10/18
5	75	M	Infarction	IV−III−V	Severe	Severe	+	+	+	–	–	–	FS	–	14/30	5/18
6	75	F	Hemorrhage	I–I–II	Moderate	Severe	+	+	+	−	–	–	FS	SP	18/30	8/18
7	72	F	Infarction	II–I–II	Moderate	Severe	+	+	+	–	–	–	FS	SP	24/30	8/18
8	76	M	Infarction	I–I–II	Severe	Severe	+	+	+	–	–	–	FS	SP, RP	10/30	7/18
9	67	F	SAH, infarction	IV−IV−V	Severe	Severe	+	+	+	–	–	–	FS	FLBP	21/30	–
10	54	M	Hemorrhage	I–I–II	Severe	Severe	+	+	+	+	–	–	FS	SP	22/30	8/18

Note

Br‐s, Brunnstrom stage; DMS, delusional misidentification syndrome; FLBP, feeling of losing a body part; FS, Fregoli syndrome; MMSE, Mini‐Mental State Examination; MS, mirror sign; RP, reduplicative paramnesia; SAH, subarachnoid hemorrhage; SP, somatoparaphrenia; USN, unilateral spatial neglect.

No patients exhibited severe visual disorders. One patient exhibited moderate bilateral hearing impairment; however, gross hearing function was preserved in all patients. Most patients exhibited severe hemiplegia and severe somatosensory impairments on the left side. All 10 DMS patients with Fregoli syndrome exhibited left unilateral spatial neglect. Nine of these 10 patients also had Pusher syndrome. Denial of paralysis was observed in two patients. Neither visual object agnosia nor prosopagnosia was observed. Assessments of cognitive function revealed a mean (±*SD*) MMSE score of 19.3 ± 5.0 (*n* = 10; range, 10–29) and a mean (±*SD*) FAB score of 9.3 ± 3.6 (*n* = 8; range, 5–17). Four patients completed the VPTA, and their ability to recognize famous and unknown faces was evaluated. All test results were within the normal range.

### 3.3 Symptomatic features of delusional misidentification

Table 2 presents the contents of delusional misidentification. The number of misidentified people varied from one to dozens. Misidentification was not corrected during hospitalization, even when medical staff or family repeatedly pointed out the error. Patients 3 and 7 exhibited DMS related to auditory information (e.g., voice, coughing), in addition to visual information. It was difficult to accurately determine the duration of symptoms because the patients were discharged from the hospital. However, all patients exhibited misidentification for at least 1.5 months rather than transient symptoms during the acute phase of the illness. Patients often misidentified staff or other patients as their acquaintances or famous people, and the contents of their delusional misidentifications remained consistent throughout the observation period (1.5–17 months).

**TABLE 2:** Contents and duration of misidentifications for patients with Fregoli syndrome

Patient	Subject(s) of misidentification	Statements	Duration (months)
1	Multiple patients and staff members	“The guy with a shaven head in the cafeteria is Mr. H senior.”	≥3
2	One nurse and one patient	“Dr. N (a familiar acupuncturist),” referring to one patient.	≥8
		“You came late,” misidentifying a nurse as his wife.	
		“Mr. S (a neighbor),” calling a roommate.	
3	Multiple patients and staff members	“That person is an announcer appearing on TV.”	≥6
		“That's •• (the patient's daughter). I can tell from the coughing,” in response to another's coughing.	
4	Multiple patients	“My daughter in Hokkaido was in the previous hospital.”	≥1
		“There are my golf friends in this hospital. I saw another in the cafeteria.”	
5	One patient	“He is my subordinate, we used to work together.”	≥4
6	One staff member and the patient's own left arm	“Dr. M,” referring to another therapist.	≥17
		“It's Dr. M’s arm,” referring to his own left arm.	
7	One staff member and the patient's own left arm	“I used to work with Mr. K,” referring to one therapist.	≥1.5
		“It is the voice of my daughter,” in response to another's voice.	
		“It's my mother's arm,” referring to her own left arm.	
8	Three staff members and the patient's own left arm	“My niece S‐chan” or “There are three Ms. *N*,” referring to staff members.	≥16
		“It is my sister's arm” or “It is my wife's arm,” referring to his own left arm.	
9	Multiple patients and staff members and the patient's own left arm	“He is the son of Ms. M, my neighbor,” referring to several patients and staff members.	≥4
		“It gets thinner from here (left forearm), and there are no fingers,” referring to their own left arm.	
10	Two staff members and the patient's own left arm	“I used to love her” and “She used to be one of the members of AKB (Japanese idol group),” referring to two staff members.	≥5

### 3.4 Lesion locations

We examined lesions that caused the Fregoli syndrome type of DMS using a case–control analysis. Because all patients with the Fregoli syndrome type of DMS exhibited right hemispheric lesions and left unilateral spatial neglect, we randomly selected 24 control patients with right hemispheric lesions and left unilateral spatial neglect but without DMS as the control group (in the following sections, these groups are referred to as the Fregoli group and non‐DMS group, respectively). The non‐DMS group consisted of 24 patients (11 women) between ages 47 and 89 years (mean ± *SD* = 71.5 ± 10.8 years). Diagnoses included cerebral infarction (*n* = 12), cerebral hemorrhage (*n* = 12), and cerebral infarction following subarachnoid hemorrhage (*n* = 0). Their mean (± *SD*) MMSE score was 23.3 ± 11.3 (*n* = 24; range, 13–30), and their mean (± *SD*) FAB score was 11.0 ± 8.4 (*n* = 20; range, 0–18). Two of the 24 patients had Pusher syndrome. There was no significant difference between the Fregoli group and the non‐DMS group regarding the sex ratio, age, diagnosis, and FAB scores. However, the incidence of Pusher syndrome was significantly higher (*p* < .001; chi‐square test) and the MMSE score was lower (*p* = .025; Mann–Whitney *U* test) in the Fregoli group.

**FIGURE 2:**
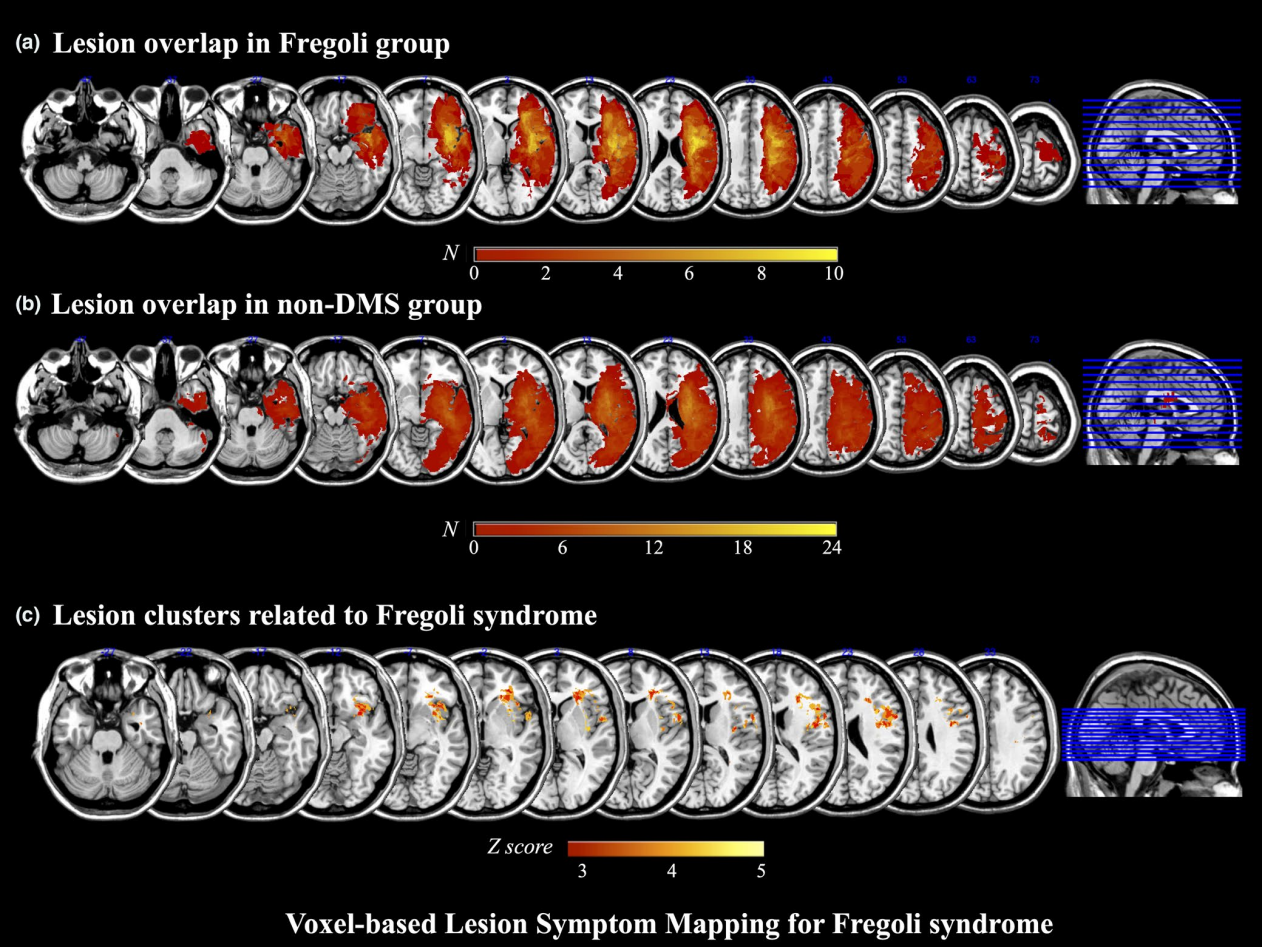
Voxel‐based lesion–symptom mapping for Fregoli syndrome. (a) Lesion overlap in the Fregoli group (*n* = 10). (b) Lesion overlap in the non‐DMS group (*n* = 24). The number of overlapping lesions is illustrated by different colors. Increasing frequency is indicated from red to yellow. Color shades indicate *z*‐scores. DMS: delusional misidentification syndrome. (c) Lesion clusters related to Fregoli syndrome. Voxel‐based lesion–symptom mapping (VLSM) comparing the lesions of the Fregoli group to those of the non‐DMS group. Color shades indicate *z*‐scores. DMS, delusional misidentification syndrome

**FIGURE 3:**
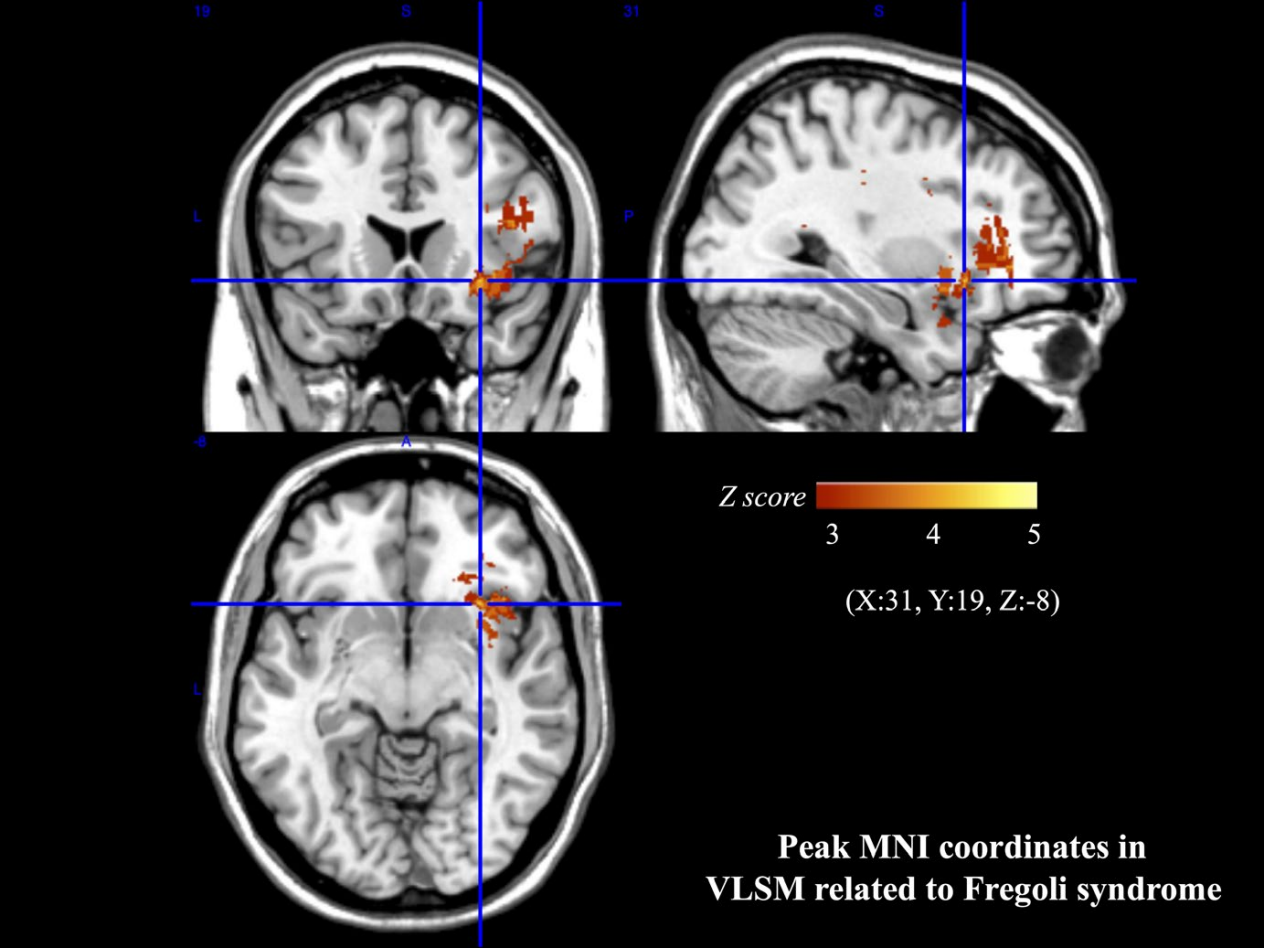
Peak MNI coordinates of VLSM for Fregoli syndrome. The peak MNI coordinates of VLSM when comparing the lesions of the Fregoli group to those of non‐DMS group were (X:31, Y:19, Z:‐8). DMS, delusional misidentification syndrome, MNI, Montreal Neurological Institute; VLSM, voxel‐based lesion–symptom mapping

MRI scans of 10 patients in the Fregoli group, MRI scans of 20 patients in the non‐DMS group, and CT scans of 4 patients in the non‐DMS group were analyzed. Figure 2 shows images of overlapping lesions in the Fregoli group (Figure 2a) and non‐DMS group (Figure 2b). Figure 2c shows VLSM comparing the lesion locations of the Fregoli group with those of the non‐DMS group. The voxelwise Liebermeister test showed that all clusters of lesion voxels specifically related to the presence of Fregoli syndrome were, in the right hemisphere, the insula, inferior frontal lobe (i.e., precentral gyrus, inferior frontal gyrus, Rolandic operculum, middle frontal gyrus), anterior temporal lobe (i.e., temporal pole, superior temporal gyrus, middle temporal gyrus, transverse temporal gyrus), olfactory cortex, and postcentral gyrus. Subcortically, the right limbic system (i.e., amygdala, parahippocampal gyrus) and basal ganglia (i.e., caudate nucleus, putamen) were also significantly associated with the presence of Fregoli syndrome. The peak MNI coordinates of VLSM when comparing the lesions of the Fregoli group with those of the non‐DMS group were at the anterior inferior portion of the right insula, including the adjacent subcortical white matter such as the extreme capsule and external capsule (Figure 3). Lesions specifically related to Fregoli syndrome were concentrated around the uncinate fasciculus that connects the inferior frontal lobe and anterior temporal lobe.

There was no significant difference between the lesion volume of the Fregoli group and that of the non‐DMS group.

## 4 DISCUSSION

### 4.1 Incidence of DMS in patients with stroke

Our results indicated that 1.1% of the patients who experienced a stroke had DMS, all of which were Fregoli syndrome. Other types of DMS, including Capgras syndrome, were not observed. This is the first study to examine the incidence of DMS among patients who have experienced a stroke; thus, we were unable to compare our results with previous studies.

According to a recent summary by Darby and Prasad (2016) among 61 patients with reported cases of delusional misidentification following brain lesions (including stroke, traumatic injury, and others), 33 exhibited DMS of people; of these patients, 17 showed signs of Capgras syndrome, 11 exhibited signs of Fregoli syndrome or intermetamorphosis, and 5 demonstrated both decreased and increased familiarity with people. Although the reported ratio of Capgras syndrome patients to Fregoli syndrome patients differed from that in our study, the incidence rates of these two disorders cannot be compared because the underlying data were collected from a review of independent case reports. A clinical bias toward focusing on Capgras syndrome may have accounted for the disproportionately large number of case reports. Although Fregoli syndrome may be overlooked as a mere cognitive deficit, Capgras syndrome can cause problems for the patient's family and close people. A time delay between stroke onset and the identification of DMS may also explain the disparate incidences (Darby & Prasad, 2016). Although our patients’ DMS symptoms were identified between 2 and 17 months after stroke onset, our observations were limited to patients in the hospital. Therefore, differences in the observation period and the familiarity of the patients with their environment (i.e., hospital vs. home) may influence the contents of delusional misidentification. It should be noted that the number of DMS cases may have been underestimated due to some constraints in the methods and conditions of this study, as described in the Limitations section below. This potential underestimation may also have affected the lesion analysis results.

### 4.2 Symptomatic features of DMS after stroke

All 10 of the patients with DMS in this study misidentified unknown people as known people, and these errors were difficult to correct. Therefore, these patients can be considered as having Fregoli syndrome (Kakegawa et al., 2016). Because the symptoms of Fregoli syndrome were consistent in content and persisted for at least 1.5 months, they cannot be explained by either confusion or delirium during the acute phase of stroke. Furthermore, because the misidentification by two patients was provoked by both auditory (e.g., voice, coughing) and visual information, the symptoms cannot be explained by agnosia limited to a specific sensory modality (e.g., prosopagnosia).

For all 10 DMS patients, the subjects of misidentification were limited to specific people; these patients did not exhibit a tendency to systematically expand the delusions to other unspecified people. Furthermore, for all patients, no sense of threat was connected with the misidentification. Instead, the patients simply showed acquaintance or mild familiarity with the misidentified people. These features of misidentification differ from those of psychotic delusions often observed in patients with schizophrenia. These characteristics differ from the classical concept of Fregoli syndrome in psychiatry. However, as there are many published reports regarding Fregoli syndrome as a type of delusional misidentification syndrome due to organic brain injury, we have used the term Fregoli syndrome in this study based on the definition put forth by Christodoulou (1986).

### 4.3 Symptomatic relationship between Fregoli syndrome and somatoparaphrenia

Among the 10 patients, Fregoli syndrome and somatoparaphrenia were observed in five patients, while three other patients were found to have somatoparaphrenia alone. Somatoparaphrenia has been likened to Capgras syndrome for one's own body because of estranged emotional attitudes toward one's paralyzed limbs (Feinberg, 2009; Feinberg & Roane, 1997). However, as was observed in the present study and in another study (Vallar & Ronchi, 2009), the reactions of patients with somatoparaphrenia to their paretic arms may be more similar to the symptoms exhibited by patients with Fregoli syndrome than to the symptoms exhibited by patients with Capgras syndrome. More than half of patients with somatoparaphrenia do not exhibit negative or estranged emotions, as is often observed with Capgras syndrome; instead, they express that the paretic arms are those of familiar people (Vallar & Ronchi, 2009). Furthermore, previous case reports of somatoparaphrenia often describe the misidentification of familiar people, even if the authors did not explicitly state that the patients had Fregoli syndrome (Berthier & Starkstein, 1987; Fotopoulou et al., 2011; Weinstein & Kahn, 1950; Weinstein, Kahn, Malitz, & Rozanski, 1954). However, little research has focused on Fregoli syndrome manifesting with somatoparaphrenia, possibly because the symptoms of somatoparaphrenia that are more obvious may overshadow those of Fregoli syndrome.

### 4.4 Causative lesions and neural substrates of Fregoli syndrome after stroke

Devinsky (2009) reviewed 29 cases of DMS, of which all patients exhibited lesions in the right hemisphere and frontal lobe (either hemisphere), suggesting that the pathology of DMS may be related to impaired self‐monitoring, ego boundaries, and deficits in the provision of emotional value and familiarity with external stimulation. Devinsky further hypothesized that release of the left hemisphere from inhibition due to damage of the right hemisphere and frontal lobe may produce excessive and incorrect explanations (i.e., confabulation) that deviate from the framework of memory and reality.

The present study indicated that the lesion sites specifically related to Fregoli syndrome were the insula, inferior frontal lobe, and anterior temporal lobe in the right hemisphere, and they were especially concentrated around the uncinate fasciculus. These results are essentially consistent with Devinsky's hypothesis. However, we further focused on the lesions in the insula and uncinate fasciculus. Activation studies of healthy participants have shown that the insular cortex is activated when processing negative emotional information that is related to faces or people (O’Doherty et al., 2003; Phillips et al., 1997; Todorov, Baron, & Oosterhof, 2008; Tsukiura & Cabeza, 2011; Winston, Strange, O’Doherty, & Dolan, 2002), as well as when processing information that is related to mentally harmful emotions in social situations (Eisenberger, Lieberman, & Williams, 2003; Sanfey, Rilling, Aronson, Nystrom, & Cohen, 2003). Moreover, voxel‐based lesion studies of degenerative diseases have demonstrated associations between the insula volume and negative emotions (i.e., disgust (Kipps, Duggins, McCusker, & Calder, 2007) and anger (Omar, Rohrer, Hailstone, & Warren, 2011). Further, previous lesion studies and recent studies using diffusion MRI tractography have shown the involvement of the uncinate fasciculus in error monitoring when learning visual object‐location associations and face‐name associations (Alm, Rolheiser, & Olson, 2016; Metzler‐Baddeley, Jones, Belaroussi, Aggleton, & O’Sullivan, 2011), and during the retrieval of proper names of people (Papagno et al., 2011). Furthermore, there is a supposed relationship between the uncinate fasciculus and social‐emotional processing such as valuation of stimuli or social reward processing (Von Der Heide, Skipper, Klobusicky, & Olson, 2013). For patients with Fregoli syndrome, lesions in the right uncinate fasciculus may impair the ability to associate information regarding social‐emotional evaluations with information related to a person's face, which is processed in the ventral stream of the occipital and temporal lobes; this impairment likely forms the basic conditions under which misidentification can occur.

The symptomatic features of Fregoli syndrome after stroke may reflect the intact function of the left hemisphere. The patients did not experience strong emotions; instead, they experienced only mild emotions to the extent that the subject of misidentification was possibly regarded as a known person without contradictions. The propositional and categorization functions of the left hemisphere require the application of existing conceptual knowledge to external stimuli, which may be inseparably accompanied to some extent by a feeling of affinity (Shobe, 2014). This mild (or fuzzy) familiarity may provide a feeling of affinity toward unknown people in unfamiliar environments, such as hospitals; however, on the contrary, it may cause a feeling of estrangement toward familiar individuals in one's own home, which would be intrinsically accompanied by strong emotions.

Recently, Darby et al. (2017) suggested that specific brain lesions for symptoms cannot be identified with superimposed images of lesion locations. Instead, they developed lesion network mapping methods to demonstrate that the right lateral inferior portion of the frontal lobe is positively correlated with delusional misidentification, while the posterior cingulate gyrus is negatively correlated with delusional misidentification. This lateral inferior portion of the frontal lobe likely includes the frontal cortices and their connections with the uncinate fasciculus. We did not observe any patients with lesions in the area of the posterior cerebral artery, including the posterior cingulate gyrus.

Concerning the complication of somatoparaphrenia in half of the Fregoli cases in this study, a previous study suggested that somatoparaphrenia may be linked to damage in the right insula (Baier & Karnath, 2008); this was also observed in the present study. Although this finding is interesting, as it suggests a common neural basis of the two symptoms, there is currently no consensus on the relationship between somatoparaphrenia and the right insula (Romano & Maravita, 2019).

### 4.5 Limitations

The present study had several limitations. First, we only evaluated patients admitted to the rehabilitation wards at two institutions, and we did not include data from patients in the acute phase of stroke or those living at home. Second, we may have overlooked delusional misidentification in patients with little spontaneous speech because the presence of DMS was identified based on patient statements. Although several structured interviews and questionnaires regarding DMS have been developed (Nagahama et al., 2007; Pagonabarraga et al., 2008; Perini et al., 2016), they are based on different methods and definitions; no standardized assessment method has been established. If a sensitive screening test for delusional misidentification had been available at the time of this study, more DMS cases may have been detected, including types of DMS other than Fregoli syndrome, including Capgras syndrome. This potential underestimation may also have affected the lesion analysis results. Future studies addressing these issues are needed to confirm our findings.

## 5 CONCLUSION

Fregoli syndrome was observed in 10 of the 874 patients who had experienced a stroke (1.1%) in the present study. Somatoparaphrenia manifesting with Fregoli syndrome was observed in five of these 10 patients, suggesting that the two conditions may share common pathological mechanisms. Somatoparaphrenia may be interpreted as Fregoli syndrome rather than Capgras syndrome related to one's own body. Careful observation may lead to increased identification of Fregoli syndrome in patients with right cerebral lesions.

## CONFLICT OF INTEREST

All authors declare no conflicts of interest associated with the contents of the manuscript.

## AUTHOR CONTRIBUTIONS

YK, OI, and TN designed the study. YK and OI recruited participants and collected clinical and neuroimaging data. YK, OI, and TN analyzed clinical data. KH analyzed imaging data. YK, OI, KH, and TN interpreted the results and wrote the manuscript.

### Peer Review

The peer review history for this article is available at https://publons.com/publon/10.1002/brb3.1829.
